# Visual pathology reports for communication of final margin status in laryngeal cancer surgery

**DOI:** 10.1016/j.jpi.2024.100404

**Published:** 2024-10-28

**Authors:** Marina Aweeda, Carly Fassler, Alexander N. Perez, Alexis Miller, Kavita Prasad, Kayvon F. Sharif, James S. Lewis, Kim A. Ely, Mitra Mehrad, Sarah L. Rohde, Alexander J. Langerman, Kyle Mannion, Robert J. Sinard, James L. Netterville, Eben L. Rosenthal, Michael C. Topf

**Affiliations:** a Department of Otolaryngology – Head and Neck Surgery, Vanderbilt University Medical Center, Nashville, TN, USA; b Department of Pathology, Microbiology and Immunology, Vanderbilt University Medical Center, Nashville, TN, USA; c Department of Otolaryngology – Head and Neck Surgery, Mayo Clinic, Rochester, MN, USA; d Department of Laboratory Medicine and Pathology, Mayo Clinic, Phoenix, AZ, USA; e School of Engineering, Vanderbilt University, Nashville, TN, USA

**Keywords:** Surgical pathology, 3D scanning, Head and neck cancer, Surgical margins

## Abstract

**Background:**

Positive margins are frequently observed in total laryngectomy (TL) specimens. Effective communication of margin sampling sites and final margin status between surgeons and pathologists is crucial. In this study, we evaluate the utility of multimedia visual pathology reports to facilitate interdisciplinary discussion of margin status in laryngeal cancer surgery.

**Methods:**

Ex vivo laryngeal cancer surgical specimens were three-dimensional (3D) scanned before standard of care pathological analysis. Using computer-aided design software, the 3D model was annotated to reflect inking, sectioning, and margin sampling sites, generating a visual pathology report. These reports were distributed to head and neck surgeons and pathologists postoperatively.

**Results:**

Fifteen laryngeal cancer surgical specimens were 3D scanned and virtually annotated from January 2022 to December 2023. Most specimens (73.3%) were squamous cell carcinomas (SCCs). Among the cases, 26.7% had final positive surgical margins, whereas 13.3% had close margins, defined as <5 mm. The visual pathology report demonstrated sites of close or positive margins on the 3D specimens and was used to facilitate postoperative communication between surgeons and pathologists in 85.7% of these cases. Visual pathology reports were presented in multidisciplinary tumor board discussions (20%), email correspondences (13.3%), and teleconferences (6.7%), and were referenced in the final written pathology reports (26.7%).

**Conclusions:**

3D scanning and virtual annotation of laryngeal cancer specimens for the creation of visual pathology reports is an innovative approach for postoperative pathology documentation, margin analysis, and surgeon–pathologist communication.

## Highlights

• Communication of final margin status in head neck surgery is crucial.
• 3D scanning and virtual annotation to create visual pathology reports is feasible.
• Visual pathology reports demonstrate margin sampling sites on oncological specimens.
• Visual pathology reports facilitated margin communication between surgeons and pathologists.

## Introduction

Total laryngectomy (TL) is used as the primary treatment of T4 laryngeal cancer, as well as in the salvage setting.^1^^,^^2^ A TL is surgical removal of the entire larynx, with the goal of completely excising malignant tissue and achieving negative tumor margins. Final margin status is important because positive margins in TL patients are a prognostic risk factor for worse local control and overall survival,^2^, ^3^, ^4^, ^5^ with a positive surgical margin increasing the risk of local recurrence at 5 years and all-cause mortality by 90%.^6^ In addition, particularly in the primary TL setting, surgical margin status is an important pathological risk factor that informs adjuvant therapy planning.^7^ After a TL is performed, the specimen is sent to the pathology laboratory for gross examination, sectioning, and microscopic evaluation. Processing cuts up the specimen and severely impacts its integrity for later use, should it be necessary.

Final margin status is communicated by the pathologist to the surgeon via the standard-of-care written pathology report, which may be limited due to lack of any visual aids. Thus, surgeons are dependent upon written descriptions in the final pathology report to identify sites of close or positive margins on complex three-dimensional (3D) specimens and determine next steps for management. In TL specimens specifically, positive and close margins are more likely to be found at the anterolateral and posterolateral soft tissue margin sampling sites,^8^ which can be composed of different tissue types such as cartilage, muscle, and thyroid gland. TL specimens must be cut open to visualize the endolarynx. The location at which the tumor is closest to the resection margin is often not in complete alignment with standard anatomical position (e.g., anterior or right) and require detailed descriptions from the prosector to ensure understanding. Thus, the relationship of these structures to the margin sampling sites can be challenging to appreciate from written descriptions alone, especially on TL specimens with large circumferential soft tissue margins.

To address this challenge, we have implemented an ex vivo 3D scanning and virtual annotation protocol for head and neck cancer surgical specimens.^9^, ^10^, ^11^ Under this protocol, resected cancer specimens are 3D scanned generating virtual 3D models. Using computer-aided design (CAD) software, a corresponding multimedia visual pathology report is created denoting inking, sectioning, and margin sampling sites on the 3D model. In the current study, we expand upon our previous published works^10^^,^^11^ to focus specifically on laryngeal cancer surgical specimens. Our goal is to explore how visual pathology reports guide interdisciplinary discussion of close and positive margins in laryngeal cancer.

## Materials and methods

### Patient population

This is a retrospective review of a virtual 3D specimen biorepository that was approved by the Vanderbilt University Medical Center Institutional Review Board (IRB #221597). Inclusion criteria were patients 18 years and older with a suspected or biopsy-proven laryngeal cancer undergoing surgical resection. Exclusion criteria were age under 18 or lack of ability to provide informed consent. Patients provided written consent before their surgery for 3D scanning and storage of the 3D specimens in a biorepository. Various types of head and neck cancer resections were scanned from January 2022 to December 2023, but this series reports specifically on the TL patients. 3D scans and visual pathology reports were created based on research team availability and surgeon and pathologist recommendations.

### 3D scanning technique

Following surgical resection, laryngeal cancer specimens are 3D scanned by a research team member before standard of care pathological analysis, as detailed in our prior work.^9^^,^^10^ A commercially available structured light 3D scanner (EinScan SP, Shining 3D, Hangzhou, China) and the corresponding software (EXScan, Shining 3D) is used to capture the 3D surface topography of fresh ex vivo surgical specimens. Specimens are taken from the operating room (OR) to the surgical pathology lab and rinsed with water and then carefully patted dry with paper towels to reduce the shine of reflective surfaces, which can interfere with the quality of the 3D scans. The specimen is then placed on the 3D scanner's turntable and the “front” surface is scanned. The specimen is flipped over 180-degrees and the “back” surface is scanned. This 3D scanning process takes on average 8 min, as demonstrated in our previously published work.^9^, ^10^, ^11^ The two virtual halves, representing the “front” and “back” surfaces, are aligned using three-point registration. Finally, the 3D scan is rendered into a watertight, virtual 3D meshed model and saved as 3MF and OBJ file types.

### Postoperative 3D specimen annotation

Laryngeal cancer specimens are typically cut open along the posterior surface through the cricoid cartilage and propped open with a cotton tip applicator, allowing for visualization of the endolarynx. Following formalin fixation, the TL specimen is examined macroscopically and prosected 1–2 days postoperatively by a member of the pathology team, most commonly a pathologist's assistant or resident physician. A member of the research team is present during pathological processing of the specimen and virtually annotates the 3D model alongside the pathology prosector. The virtual 3D scanned model is imported as a 3MF file type into a CAD software (Meshmixer, Autodesk Inc., San Rafael, CA) for virtual inking, sectioning, and processing.

In CAD software, the 3D model is “inked” using the “Paint Vertex” virtual paintbrush. An inking color key is created to denote the different anatomical surfaces of the TL specimens. Black inking is used to mark the “right” side of the specimen, whereas blue inking specifies the “left”. White shaded rectangles are used to represent the length, width, and location of perpendicular sections. Purple shading is implemented to denote sites of shave margins. The virtual paintbrush feature also functions to create alphabet labels for each margin sampling site to correspond with the written pathology report. Sectioning, inking, and labeling are conducted in real-time to reflect the prosector's processing of the real surgical specimen.

After completion of the visual pathology report, the CAD file is saved and exported as a 3MF file type. In Microsoft PowerPoint (Microsoft Corporation, Redmond, WA), interactive virtual models of the 3D scan and visual pathology report are manufactured by importing the respective 3MF file types. The 3D models are animated to complete two 360-degree rotations on the vertical and horizontal axes using the “animation” feature. A virtual key is created using text and symbols to detail the different types of inking and sectioning. The 2D photos taken of the specimen before processing are also imported into the PowerPoint slide. Lastly, the PowerPoint slide is saved and exported as a “full HD” MP4 video file type to create a 20 s video featuring the animated 3D scan and specimen map models.

### Multidisciplinary communication

Final visual pathology reports are distributed via email to the attending surgeons and pathologists. After the final pathology report is drafted, the surgeon and pathologist use the visual pathology reports to discuss close or positive surgical margins and reconcile separate defect margins (if taken) for final margin status via teleconference (Zoom Video Communications, San Jose, CA) meetings, email, or phone discussion. Additionally, visual pathology reports are shared by the pathologist and head and neck surgeon during virtual multidisciplinary head and neck cancer tumor board meetings using Microsoft Teams (Microsoft Corporation, Redmond, WA) to discuss close or positive margin sites.

Cases were categorized based on final margin status as follows: (1) Positive: Tumor microscopically present at the inked border of the main specimen resection or in the separate defect-driven margin specimens if the surgeon used a tumor bed-based approach for margin analysis. (2) Close: Negative margins on the main specimen but with a clearance of <5 mm. (3) Negative: Negative margins on the main specimen and negative separate defect margin specimens if the surgeon used a tumor bed approach, with adequate clearance >5 mm of the closest margin. Margin status is established by a combination of gross measurements, using a ruler on the cut surface of the specimen, and by microscopic measurements, which are obtained by using a translucent ruler overlayed on top of the slide or by digital measurement photographically.

## Results

### Patient population

From January 2022 to December 2023, visual pathology reports were generated for 15 TL specimens. The patients were predominantly male (86.7%, *n* = 13), with a median age of 66 (range 44–79). Six patients (40%) had history of radiation therapy and underwent salvage TL. Most surgical specimens were excised for squamous cell carcinoma (SCC) (73.3% *n* = 11) followed by chondrosarcoma (13.3%, *n* = 2), and adenoid cystic carcinoma (ACC) (13.3%, *n* = 2). The most common primary tumor site was supraglottic (60%, *n* = 9). Several specimens also included structures such as additional pharyngeal mucosa (40%, *n* = 6) and thyroid gland (46.7%, *n* = 7). The majority of patients (60%, *n* = 9) were pathological T4a. Additional information for all cases can be found in Table 1.

**Table 1:** Case characteristics.

Case No.	Surgical specimen	Pathology	Primary site	pT staging	pN staging	Margin classification	Post-operative communication
1	Total larynx and hypopharynx	SCC	Supraglottic	T4A	3B	Negative	NO
2	Total larynx	Chondrosarcoma	Thyroid cartilage	T1	N0	Close	YES – Email discussion between surgeon and pathologist
3	Total larynx and L hemithyroid	SCC	Supraglottic	T3	3B	Negative	NO
4	Total larynx	SCC	Supraglottic	T4A	2B	Negative	NO
5	Supracricoid larynx	Chondrosarcoma	Thyroid cartilage	T1	X	Negative	NO
6	Total larynx	SCC	Glottic	T4A	N0	Negative	NO
7	Total larynx and total thyroid	SCC	Supraglottic	T3	N3B	Negative	NO
8	Total larynx and partial pharynx	SCC	Supraglottic	T4A	N3B	Close	NO
9	Total larynx and R base of tongue	SCC	Supraglottic	T3	N0	Positive	YES – 3D specimen map referenced in final pathology report
10	Total larynx and R hemithyroid	Adenosquamous carcinoma	Glottic	T4A	N0	Negative	YES – Email discussion between surgeon and pathologist
11	Total larynx, hypopharynx, and L hemithryoid	SCC	Supraglottic	T4A	N0	Close	YES – 3D specimen map used in multidisciplinary tumor board
12	Total larynx and total thyroid	SCC	Supraglottic	T3	N0	Negative	YES – 3D specimen map used in multidisciplinary tumor board
13	Total larynx, hypopharynx, and R hemithyroid	SCC	Supraglottic	T4A	N0	Positive	YES – Zoom discussion between surgeon and pathologist
14	Total larynx, total pharynx, and R hemithyroid	Adenoid cystic carcinoma	Subglottic	T4A	N0	Positive	YES – 3D specimen map used in multidisciplinary tumor board
15	Total larynx, partial pharynx, and anterior esophagus	Adenoid cystic carcinoma	Subglottic/ Trachea	T4A	N0	Positive	YES – 3D specimen map referenced in final pathology report

Four cases (26.7%) had final positive surgical margins. Case #9 was a salvage TL for a recurrent pT3N0 supraglottic SCC with the base of tongue soft tissue margin anterior to the epiglottic remnant positive for invasive tumor. The patient declined adjuvant re-irradiation and will continue to receive close cancer surveillance care. In Case #13, the patient received a primary TL for a pT4aN0 supraglottic well differentiated SCC with verrucous features. Tumor was present at the posteroinferior soft tissue resection margin, was 3 mm from the inferior hypopharyngeal mucosal margin, and 2 mm from the left superior hypopharyngeal margin. The patient had significant medical comorbidities, including gastric cancer, and declined further therapy and transitioned to hospice. Case #14 was a primary TL for a pT4aN0 subglottic, stage III, laryngeal ACC. Tumor was present at the anterior pre-laryngeal soft tissue margins. The patient is currently receiving adjuvant radiotherapy (RT) for the positive margin. Lastly, Case #15 was a primary TL for a pT4aN0 subglottic ACC, with invasive tumor at the anterior and posterior inferior tracheal margins. The surgeon was unable to resect further trachea and still create a stoma. The patient is scheduled to receive adjuvant RT.

Two cases (13.3%) had close margins on final pathology. Case #2 was a TL for recurrent chondrosarcoma, which was present 1 mm from the inferolateral anterior soft tissue margin. At the time, the patient was recommended for careful observation of this close margin. In Case #11, the patient received a salvage TL for a recurrent pT4aN0 supraglottic SCC. Final pathology revealed invasive carcinoma 2 mm from the left anterolateral soft tissue margin. The patient had a history of significant radiation toxicity, so close surveillance was recommended rather than further adjuvant therapy.

### Postoperative visual pathology report

**Fig. 1:**
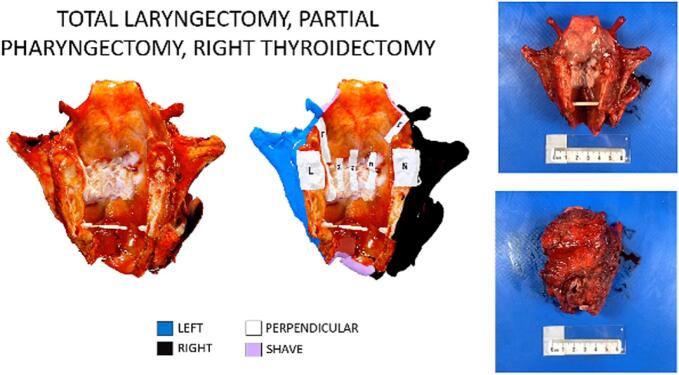
Multimedia visual pathology report created and shared via email with head and neck surgeons and pathologists for Case #13: a total laryngectomy, partial pharyngectomy, and right thyroidectomy specimen with final positive margins.

**Fig. 2:**
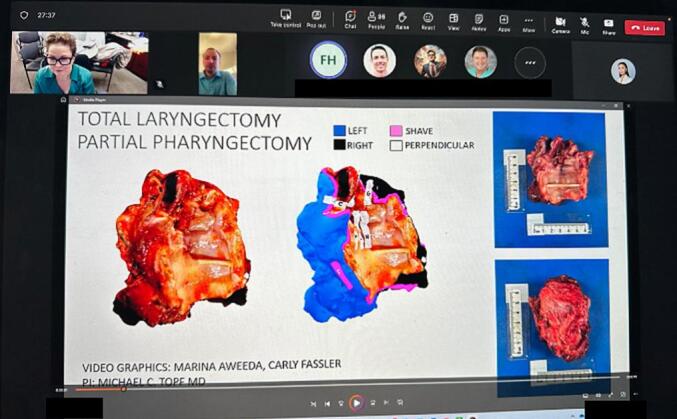
Visual pathology report presented during multidisciplinary tumor board for Case #11, a pT4aN0 supraglottic squamous cell carcinoma total laryngectomy specimen with close final margins. Representatives from head and neck surgery, pathology, radiology, medical oncology, and radiation oncology were present to discuss margin status and recommendations for adjuvant therapy.

Visual pathology reports were created postoperatively to document the inking, cutting, and sections taken for histology. These reports were then electronically distributed to the head and neck surgeons and pathologists on the same day as pathological gross examination, before release of the final pathology report (Fig. 1). When the written pathology reports were finalized, the visual pathology reports were used to facilitate postoperative communication of margins in cases with close or positive margins (85.7%, *n* = 6). The visual pathology reports were utilized in multidisciplinary tumor board (20%, *n* = 3) (Fig. 2), email (13.3%, *n* = 2), and teleconference (6.7%, *n* = 1) discussions, and were referenced in the final pathology reports (26.7%, *n* = 4). Two illustrative cases are described in detail.

#### Case #1

A 77-year-old male with SCC of the larynx with verrucous features underwent a TL, partial pharyngectomy, right thyroidectomy, and bilateral neck dissection with primary closure. Intraoperatively, frozen section analysis (FSA) margins were sent on the post-cricoid, inferior pharyngeal, superior pharyngeal, and base of tongue margins, which were all negative for malignancy. The specimen was 3D scanned on the day of surgery, and the visual pathology report was completed the following day.

**Fig. 3:**
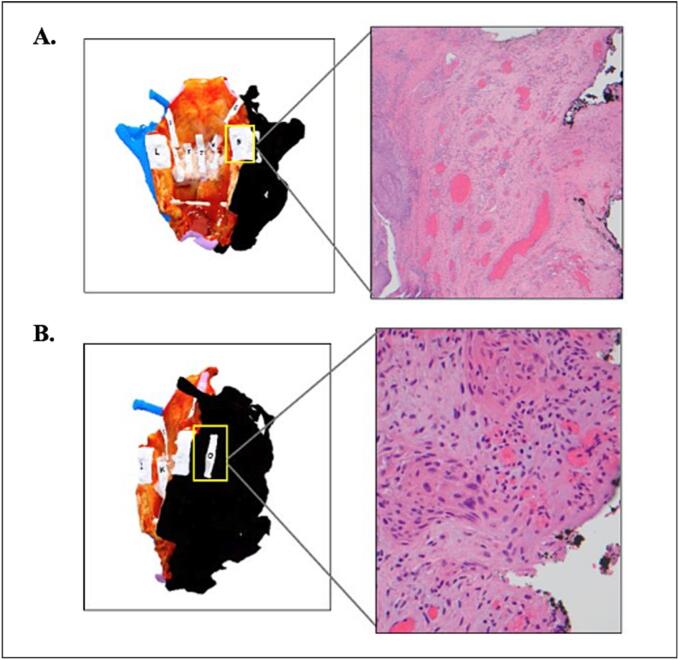
Three-dimensional (3D) images of the posterior (A) and right (B) aspects of a total laryngectomy specimen for Case #13, Illustrative Case #1. Inlayed two-dimensional (2D) images (hematoxylin and eosin, H&E), corresponding to the alphabetic gross tissue cassettes, highlight sampling site “N” where the tumor is close (2 mm) to the inked margin, but negative (4× magnification, top-right). A nearby separate section, “O,” showed tumor focally present at the inked surface, constituting a positive margin (20× magnification, bottom-right).

**Fig. 4:**
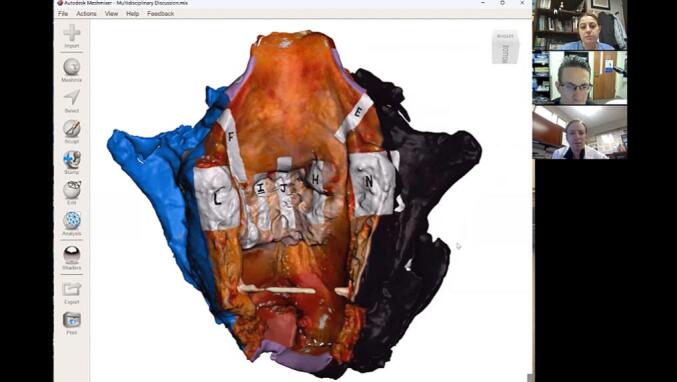
Visual pathology report discussed by surgeon and pathologist using Microsoft Teams teleconference for Case #13, Illustrative Case #1. The three-dimensional (3D) total laryngectomy specimen is presented within the computer-aided design software in the center of the image. A member of the research team rotates the virtual 3D specimen as the surgeon and pathologist discuss sites of close and positive margins.

The visual pathology report multimedia presentation was shared via email with the head and neck surgeons and pathologists involved in the case. The surgeon and pathologist met via teleconference to discuss the findings using the visual pathology report (Fig. 3). The final pathology report revealed a pT4aN0 well differentiated keratinizing type SCC with verrucous features arising in the left supraglottis and involving the bilateral true and false vocal cords, with invasion through the cricoid cartilage. The tumor was microscopically present at the right posteroinferior soft tissue resection margin, and close to two other lateral soft tissue margins. In the comments section of the pathology report, the pathologist stated: “Invasive carcinoma is close to two peripheral margins (left lateral, 1mm and right posterior, 2mm) and present at the inked margin right posterolaterally. These were discussed with Dr. [Surgeon Name] including review of the 3D specimen maps and constitute a focal positive margin.” (Fig. 4).

#### Case #2

**Fig. 5:**
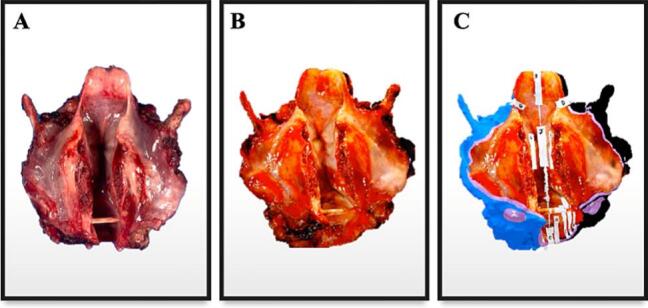
A. Two-dimensional picture of fresh surgical specimen. B. Three-dimensional (3D) model of surgical specimen after 3D scanning. C. 3D model after virtual annotation using computer-aided design.

A 55-year-old male with subglottic laryngeal ACC underwent a TL, total pharyngectomy, hemithyroidectomy, left neck dissection, and reconstruction with a radial forearm free flap. Intraoperatively, the inferior tracheal margin was submitted for FSA, and was negative for malignancy. The specimen was 3D scanned on the day of surgery and virtually annotated the following day during pathological processing (Fig. 5). The visual pathology report was shared via email with the head and neck surgeons and pathologists. The final pathology report revealed a pT4a pN0, grade III (solid pattern), ACC arising in the subglottis and superior trachea, with invasion through the anterior commissure, subglottis, true vocal cords, and thyroid and cricoid cartilages. There was vascular invasion with tumor microscopically present at the anterior soft tissue and inferior tracheal margins, resulting in a positive final margin status.

**Fig. 6:**
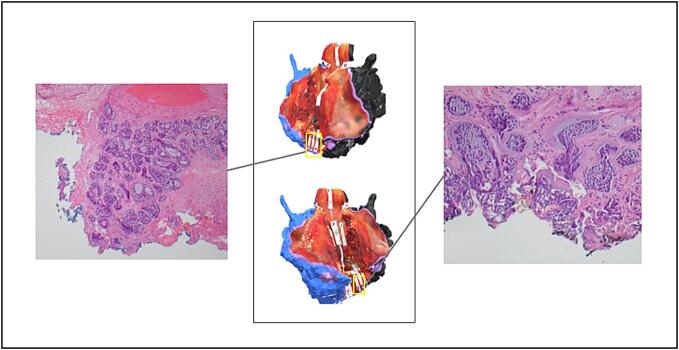
Three-dimensional (3D) images of the inferior posterior aspect of a total laryngectomy specimen corresponding with Case #14, Illustrative Case #2. Yellow rectangles highlight anterior margin sampling sites “P” and “R.” Inlayed two-dimensional (2D) images (hematoxylin and eosin, H&E), show the tumor abutting the blue-inked left margin (left image, 10× magnification) and black-inked right margin (right image, 20× magnification). Thermal effect on the tissue is present at the inked surface, further supporting the reported positive margin status. (For interpretation of the references to color in this figure legend, the reader is referred to the web version of this article.)

This case was presented during multidisciplinary head and neck tumor board, with representatives from surgical, medical, and radiation oncology, as well as radiology and pathology present. The pathologist used the “screen share” feature on Microsoft Teams to display the visual pathology report and was able to directly demonstrate where the positive margin sites were on the 3D model, correlating them with the histological analysis (Fig. 6). The head and neck surgeon discussed the inferior tracheal margin and confirmed that he had resected an additional tracheal ring intraoperatively which superseded the inferior border of the specimen and was negative for malignancy on FSA, thus confirming the true inferior tracheal margin was negative. However, the anterior soft tissue margin was positive. The head and neck surgeon, pathologist, and radiation oncologist discussed the case using the visual pathology report and made the final recommendation of adjuvant RT.

## Discussion

In this proof of concept study, we aimed to assess how visual pathology reports of laryngeal cancer specimens affect postoperative communication of margin status between head and neck surgeons and pathologists. We generated visual pathology reports for 15 laryngeal cancer specimens. In cases where close or positive margins were identified, the visual pathology reports were used to facilitate communication between the head and neck surgeons and pathologists. These reports were utilized in various communication platforms, including multidisciplinary tumor boards, email exchanges, and teleconference discussions, as well as incorporation into the final pathology report. Moreover, the visual pathology reports served as permanent visual records of the specimen grossing process for all cases.

The visual pathology reports were used clinically in all 15 cases. Four cases had positive final margins and two cases had close surgical margins. At our institution, final pathology reports are typically released within 2 weeks following the surgical resection. Specimens are stored for at least 2 weeks following release of the written final report before being released for disposal. However, the specimen integrity is often significantly altered after the initial prosection, limiting the utility of viewing it for clarification by the pathologist. In this study, the visual pathology reports virtually replicated the original specimen and provided a permanent record of margin inking and tumor sampling, enabling the pathologist to see the specimen essentially as it arrived to gross room. This visual report was additionally beneficial in supplementing the surgeon-pathologist discussion. Our pathologists found subjective benefit in the models for correlation between the written gross description and the glass slides, as the annotations are far more specific than the combination of the description and still (2D) photographs conventionally. In the four cases with positive surgical margins, the surgeons and pathologists were able to pinpoint the specific margins of interest on the 3D TL models. These visual pathology reports served as a focused visual aid that followed the specimen through resection, gross processing, and final reporting.

From the pathologists' perspectives, the 3D images allowed for the ideal photo documentation of the specimens. They were most useful in the case of close or positive margins, particularly for reconciling where the margins are exactly located on the specimen. In cases of separate “defect” margins, knowing with more certainty whether (and how) they aligned (or not) with the sites of tumor was also beneficial. With the 3D images, in addition to visualizing the location of the tumor and margins sampling, it allows one to rotate the image to the optimal viewing frame to show the anatomy, specimen details, and specific margins.

Laryngeal cancer specimens pose unique challenges in gross pathological examination due to their complexity. Because TL specimens must be cut open to visualize the endolarynx, the location at which the tumor is closest to the resection margin is often not in complete alignment with standard anatomical position (e.g., anterior or right) and require detailed descriptions from the prosector to ensure understanding. A retrospective review of 70 patients undergoing TL for laryngeal SCC found that positive margins most commonly involved the anterolateral and posterolateral soft tissues (19% and 11%, respectively), followed by the postcricoid and superior (7% and 1.4%, respectively) mucosal margin sampling sites.^8^ These sites can consist of cartilage, muscle, mucosa, and thyroid gland. Without visual representation, it may be challenging to understand the margin sampling sites and their relationship with surrounding structures. These complexities underscore the importance of careful margin assessment and clear delivery of findings in laryngeal cancer specimens.

In laryngeal cancer surgery, pathological assessment of TL specimens provides valuable insights regarding tumor grading and staging, depth of invasion, and surgical margin status.^12^ 3D scans obtained by this process are stored in the same file locations as the standard 2D images captured during specimen processing, and are readily accessible by the pathologists signing out the final report. Given the pathologist reviewing the slides for a case is rarely involved in the gross examination of the specimen itself, we find that the 3D representations are an excellent substitute which enhance the sign out process. This is especially important in complex specimens of the head and neck, such as composite resections and total laryngectomies, among others. Additionally, this technology allows a near-perfect representation of the specimen to exist long after the disposal of the specimen, in accordance with College of American Pathologists guidelines for retention of gross tissue (14 days following issuance of the final report.) This enables examination of the specimen for usage in medical education, tumor board discussions, and more.^13^

Based on the findings presented in the final pathology report, the clinical care team determines the next steps for management, including need for adjuvant therapy.^14^^,^^15^ Accurate communication of this information is essential, but is limited to text descriptions in the standard-of-care written pathology report. One study comparing clinical comprehension of written pathology reports with pathologist intent found that surgeons misunderstood pathologists' reports 30% of the time.^16^ Although surgical experience reduced this discrepancy, it did not resolve the problem. Similarly, Talmon et al. found that miscommunication of intraoperative frozen section results between surgeons and pathologists happened about 9.6% of the time.^17^ The authors in both studies emphasized that this was a communication challenge and a misinterpretation of intended meanings, rather than an intrinsic fault of either party to understand and describe the disease processes.

Several studies have emphasized the wide variation in descriptive language used in the diagnosis section of surgical pathology reports, leading to potential miscommunication and uncertainty among non-pathologist faculty interpreting these reports.^18^, ^19^, ^20^ Whereas methods such as tumor boards, face-to-face consultations, and phone calls may enhance communication between pathologists and head and neck surgeons,^20^ their implementation is inconsistent across institutions. Limited data exist regarding how surgical margin status in head and neck cancer resections is relayed between surgeons and pathologists. Given the complexity of this anatomic subsite and the prognostic significance of surgical margin status, we aimed to introduce a multimedia visual tool to supplement traditional written pathology reports and faciltate communication of close and positive margins in complex laryngeal cancer specimens.

The feasibility and practicality of 3D scanning have been demonstrated in prior studies by our team and others. Implementation of 3D scanning and virtual annotation in our gross lab has been successful, with an additional 8–10 min of processing time deemed acceptable by both pathologists and surgeons at our institution.^9^^,^^21^ Previously, we reported that key stakeholders at our institution agreed that 3D scanning and virtual specimen annotation improved communication between head and neck surgeons and pathologists.^9^ Furthermore, Miller et al. showed that specimen mapping of head and neck resections creates a permanent visual record of the specimens and their margin sampling sites, which may serve as a valuable tool both intra- and postoperatively among the multidisciplinary care team.^10^^,^^11^ Saturno et al. and Yun et al. from the Mt. Sinai group have also demonstrated successful protocols for 3D scanning of surgical specimens and intraoperative communication of margin analysis, highlighting both the feasibility and reproducibility of these techniques across different institutions.^22^, ^23^, ^24^ An editorial review by Brandwein-Weber et al. argued that intraoperative 3D specimen scanning holds promise for enhancing communication of surgical margin status, emphasizing the need for further multi-institutional investigations to validate these techniques with actual showing improvements in patient care and outcomes.^25^

When considering the widespread implementation of 3D specimen scanning and visual pathology reports, it is important to acknowledge its limitations. A signficiant bottleneck in our workflow has been the manual digital markup of the 3D models to create visual pathology reports. For complex specimens like laryngeal cancer resections, this step may take up to 1 h per specimen. To address this, we have assigned a dedicated team member to digitally annotate the specimen, whereas the prosector conducts the gross examination. Whereas it is still unclear if the additional time and cost associated with personnel performing 3D scanning, postprocessing, and digital annotation are justified by the clinical benefits, we see potential in this technique for improving communication, especially in scenarios where access to pathology labs may be limited for surgical staff. Furthermore, we use a commercially available 3D scanner, priced at approximately 3000 USD, along with free CAD software for specimen mapping. In our experience, the 3D scanning and CAD skills necessary for this protocol are not overly complicated and can be taught to individuals with a range of experience, including beginners.

Future directions for this research involve developing specific software tailored for the annotation of virtual 3D specimen models, with the goal of streamlining the process and making it more widely accessible. We envision a custom annotation software that can be integrated into the standard grossing workflow, so the prosector grossing the specimen can virtually annotate the 3D specimen model in real time as they process the specimen. We believe that this is an important next step for widespread implementation as it compliments the existing workflow and eliminates the necessity for dedicated research staff to perform the annotation. Most importantly, like any novel technology implemented, we will ultimately need to demonstrate value for this approach for our patients and providers. Surgeon–pathologist communication is challenging to study, but we believe it is imperative to explore and improve upon, especially in head and neck cancer. Moving forward, we will perform a prospective, survey-based study with a larger sample size to assess the qualitative improvement in surgeon–pathologist communication. Ultimately, a larger goal of this research is to integrate the visual pathology reports directly into the patient's electronic medical record, so that all members of the care team can have easy access to this information.

## Conclusion

Visual pathology reports of laryngeal cancer resections can be used to facilitate postoperative communication of close and positive margins between head and neck surgeons and pathologists. Visual pathology reports may serve as a visual aid to supplement the written pathology report, creating a permanent record of the specimen grossing process.

## Funding

This work was supported by a Vanderbilt Clinical Oncology Research Career Development Program (K12 NCI 2K12CA090625-22A1); 10.13039/100007208Vanderbilt-Ingram Cancer Center Support Grant (P30CA068485); and AHNS/AAO-HNSF Young Investigator Combined Award.

## Ethical approval

This study was approved by the Vanderbilt University Medical Center Institutional Review Board (IRB #221597).

## Declaration of competing interests

No conflicts of interest to disclose.

The following are the supplementary data related to this article.

Supplementary video 1 Visual pathology report of a total laryngectomy, pharyngectomy, and right thyroidectomy patient. Supplementary video 1

Supplementary data to this article can be found online at https://doi.org/10.1016/j.jpi.2024.100404.

## Data Availability

The authors confirm that the data supporting the findings of this study are available within the article and its supplementary materials and are available from the corresponding author, MCT, upon reasonable request.
